# Sirt-1 Regulates Physiological Process and Exerts Protective Effects against Oxidative Stress

**DOI:** 10.1155/2021/5542545

**Published:** 2021-03-23

**Authors:** Lei Liu, Guangyuan Xia, Peifan Li, Yiming Wang, Qian Zhao

**Affiliations:** ^1^Department of Mental Health and Psychiatry, The Second Affiliated Hospital of Soochow University, Suzhou, Jiangsu, China 215006; ^2^Department of Psychiatry, Affiliated Hospital of Guizhou Medical University, Guiyang, Guizhou, China 550025; ^3^Department of Psychiatry, Zaozhuang Mental Health Center, Zaozhuang, Shandong, China 277103; ^4^College Students' Mental Health Education and Counseling Center, Guizhou Medical University, Guiyang, Guizhou, China 550004; ^5^Department of Nuclear Medicine, General Hospital of Ningxia Medical University, Ningxia, Ningxia Hui Autonomous Region, China 750004

## Abstract

**Background:**

Recent studies suggest a correlation between the reduced Sirt-1 expression with Alzheimer's diseases (AD) and depression, respectively, suggesting a possible pathogenic role of the altered Sirt-1 expression in neuronal degenerative diseases, such as AD and depression. However, the molecular mechanisms underlying how Sirt-1 reduction impairs neuronal functions remain unknown.

**Methods:**

We used the SK-N-SH neuroblastoma cells to study the role of Sirt-1 expression on physiological roles in neuronal cells. Gain of Sirt-1 was achieved by transiently transfecting Sirt-1 expression plasmid. Sirt-1-specific shRNA was used to elucidate the role of Sirt-1 loss of function. CCK-8 (Cell Counting Kit-8) assay and flow cytometry were used to evaluate cell proliferation. Semiquantitative western blotting was used to detect relative protein levels. A further luciferase reporter gene assay was employed to examine the effect of Sirt-1 expression on the transcriptional activity of p53. RT-qPCR was used to determine the mRNA levels of p21, Bax, and Bcl-2, which were the downstream target genes of p53.

**Results:**

Sirt-1 suppressed the p53 downstream gene p21 transcription, while shRNA-mediated Sirt-1 knockdown resulted in a significant increase in p21 expression, implying a possibility that Sirt-1 promotes neuron proliferation through suppressing p53 transcriptional activity. The mRNA and protein levels of p53 were not affected by the altered Sirt-1 expression, suggesting that Sirt-1 regulates the transcriptional regulatory activity of p53 rather than p53 expression. Indeed, we further confirmed that Sirt-1 appeared to inhibit p53 transcriptional activity by attenuating its acetylation and resulted in a decrease of p53's binding to the p21 promoter. Overexpressed Sirt-1 scavenged reactive oxygen species (ROS) production in SK-N-SH with H_2_O_2_. Knockdown of Sirt-1 presented opposite effect; the addition of EX527 (Sirt-1 inhibitor) increased ROS accumulation.

**Conclusions:**

Oxidative stress induces Sirt-1 in neuron cells, and Sirt-1 promotes proliferation in SK-N-SH cells, which protects them from oxidative stress-induced cell death, potentially via suppressing the transcriptional activity of p53. These results provide a molecular explanation underlying how the reduced Sirt-1 potentially causes the AD and depression-related diseases, supporting the idea that Sirt-1 can possibly be used as a diagnostic biomarker and/or therapeutic drug target for the AD and depression-related diseases.

## 1. Introduction

Silent mating type information regulation 2 homolog 1 (Sirt-1), which is a member of the sirtuin family, exerts stress-protecting effects against inhibition of cell cycle entry and induction of apoptotic cell death via transcriptionally regulating gene expression [1]. Sirt-1 is one of the nicotinamide adenine dinucleotide- (NAD^+^-) dependent histone deacetylases and is discovered to be a critical component of seven homologous proteins regulating cell functions through chromatin remodeling/histone deacetylation [2–4]. In these manners, Sirt-1 plays critical roles in many human physiological functions, including DNA repair, aging, gene expression, and apoptosis [5]. In mouse C2C12 myoblasts, Sirt-1 was found to inhibit the differentiating processes and expression of myogenin, which resulted in the proliferating inhibition and inmaturation of satellite cells [6, 7]. Oberdoerffer and colleagues reported that, in mammalian cells, including in neurons, Sirt-1 was triggered by DNA damage and then redistributed to DNA breaks for repairmen, functioning as a transcriptional factor [8]. Notably, Sirt-1 was also found to be a neuroprotective molecule, which protected neurons against oxidative stress-induced cellular damage and stressful perturbations in both acute and chronic neurological diseases, including AD [9, 10]. In animal models of AD and Huntington's disease, overexpression of Sirt-1 abrogated neuronal degeneration and nonapoptotic and apoptotic cell death and indicated the tight association of Sirt-1 to physiological processes in neurons.

Sirt-1 interacts with several target substrates, including forkhead box O (FOXO) family members and p53 [11–13]. It was shown that in mammalian cells, Sirt-1 appeared to control the cellular response to stress by interacting with the FOXO family of forkhead transcription factors and thus exerting protective effects against oxidative stress-induced cellular damage [14]. By exerting deacetylating activity, Sirt-1 inactivates p53 and thus regulates cell survival and proliferation [13]. Suppression of Sirt-1 resulted in hyperacetylation of p53, which prevented its binding to Mdm2, resulting in cell cycle arrest and apoptosis [13]. The cyclin-dependent kinase (CDK) inhibitor p21 is a critical regulator in the cell cycle checkpoint resulting in the inhibition of G_1_ progression and induction of senescence by blocking the activity of CDKs, without knowing how cell cycle is blocked [15, 16]. p21, which is one of the most well-known p53 target genes, is tightly transcriptionally regulated by deacetylated p53 and results in p53-dependent G_1_ arrest after DNA damage [17]. By considering the regulation of Sirt-1 on FOXO and p53, we hypothesized that Sirt-1 might exert protective effects in neurons by deacetylating FOXO and p53.

Oxidative stress is a state which might either generates or accumulates the levels of cellular reactive oxygen species (ROS) [18]. Increasing ROS may dysfunction mitochondrial, damaging lipids, proteins, and DNA, which are consistently observed in AD subject autopsy brains [19]. In recent years, oxidative stress is recognized as a central factor in major depressive disorder (MDD) and AD. Oxidative stress-induced ROS accumulation causes brain damage and triggers specific antioxidant defense, including increasing the expressing level of amyloid-*β* and accumulation of hyperphosphorylated tau, leading to complex pathological cascades culminating in AD [20–22]. In the rat brain, activation of Sirt-1 inhibited p53 acetylation and ROS production, which was further promoted by the addition of EX527, a Sirt-1 inhibition, which indicated that Sirt-1 might exert a protective effect against oxidative stress-related AD, by deacetylating p53 and thus scavenged ROS accumulation [23].

Here, we aimed to investigate whether Sirt-1 might protect neuronal cells against oxidative stress by regulating FOXO or p53. SK-N-SH neuroblastoma cells were employed to figure out the roles of Sirt-1 expression on physiological roles in neuronal cells. This study determined for the first time that Sirt-1 might regulate p53 depending on its deacetylation activity and scavenge ROS induced by oxidative stress, demonstrating the importance of Sirt-1 and oxidative-induced ROS in the progression of AD and relative diseases.

## 2. Material and Methods

### 2.1. Cell Culture and Treatment

SK-N-SH cells (human neuroblastoma cell line) and SK-N-AS (p53-null cell line) were purchased from the Chinese Academy of Sciences (Beijing, China). As described by Huang et al. [24], SK-N-SH cells were cultured in Dulbecco's modified Eagle's medium (DMEM, Gibco, USA) at 37°C in a 5% CO_2_ humidified atmosphere, with 10% fetal bovine serum (FBS, Gibco, USA) and 1% penicillin. When cells were70-80% confluent, Opti-MEM was used with 2 h prior to transfection.

### 2.2. siRNA Transfection

We followed the methods of Kobayashi et al. [25]. The Sirt-1-specific si-Sirt-1 (GenePharma, China) was synthesized to knockdown the expression of Sirt-1 in cells. The sequence of si-Sirt-1 and siRNA-NC were provided by GenePharma (China). siRNA transfection was conducted according to the manufacturer's instructions. After transfection for 72 h, total protein and RNAs were prepared for western blotting analysis and qPCR, respectively.

### 2.3. Western Blotting

Cells were lysed, and BCA assay (Beyotime Biotechnology, China) was used for detecting the protein concentration. Western blotting was conducted by the methods of Xu et al. [26]. Primary antibodies were as follows: Sirt-1, Abcam, #ab220807; FOXO1, Abcam, #ab39670; p53, Abcam, #ab26; p21, Abcam, #ab109520; p53 (acetyl K382), Abcam, #ab75754; p53 (acetyl K120), Abcam, #ab78316. *β*-Actin was used as an internal control. The chemiluminescence detection kit ECL (Millipore, USA) was used to detect the bound antibodies.

### 2.4. Luciferase Reporter Assay

To evaluate the interaction between Sirt-1 and the Nanog 3′-UTR, the dual-luciferase reporter assay (Promega, USA) was conducted. The Nanog 3′-UTR and the mutant Nanog 3′-UTR were cloned and sequenced. We followed the methods of Xu et al. [26]. After transfection for 48 h, luciferase activity was detected and normalized with the Dual-Luciferase Reporter Assay System (Beyotime Biotechnology, Haimen, China).

### 2.5. CCK-8 Assay

We followed the methods of Wang et al. [27]. CCK-8 assay was conducted by using Cell Counting Kit-8 (AbMole Bioscience, USA). Cells were seeded into a 96-well plate (5.0 × 10^3^/well) and exposed to si-RNA or not for 1 to 5 days. Then, 10 *μ*L CCK-8 was added and cells were further incubated for 4 h. A microplate absorbance reader (Bio-Rad) was used to detect cell viability at 490 nm.

### 2.6. Apoptosis Analysis

Apoptosis analysis was conducted with the annexin V-FITC apoptosis assay kit (Abcam, Britain) according to kit instruction, as described by Qi et al. [28]. Cells (5 × 10^5^‐1 × 10^6^/mL) were suspended in 200 *μ*L binding buffer; 10 *μ*L annexin V-FITC were added, and cells were incubated in the dark, 4°C for 30 minutes. Then, 300 *μ*L binding buffer and 5 *μ*L PI were added and cell apoptosis was tested on the machine within 1 hour.

### 2.7. Colony Formation in Soft Agar

As described by Xu et al. [26], 1 × 10^3^ cells were mixed with 0.3% low melting agar, supplemented with DMEM (including 10% FBS). Then, cells were plated on a low melting agar-coated 6-well plate (0.6%) and incubated at 37°C for 15 days. Cells were stained with crystal violet (0.05%, 0.2 mL/well) for 30 min, at 37°C. The numbers of positive colonies (>8 cells/colony) were counted. The test was repeated twice.

### 2.8. EdU (5-Ethynyl-2′-Deoxyuridine) Staining

We followed the methods of Zeng et al. [29]. Cells (2° × °10^5^/well) in a 6-well plate were supplemented with DMEM containing 50°*Μ*m EdU (RiboBio, Guangzhou, China) for 2 hours. The cells were washed (4°C) and fixed with 4% paraformaldehyde (at room temperature) for 10 min. EdU immunostaining was conducted with Apollo staining reaction buffer. Stained cells were observed under the fluorescence microscope (Olympus, Melville, NY).

### 2.9. Cell Cycle Analysis

As described by Qi et al. [28], cell cycle analysis was conducted with the Cell Cycle Analysis Kit (BioVision, USA) according to kit instruction. Cells were suspended and washed with PBS three times. Then, a cell pellet was fixed with 70% alcohol (ice-cold) at 4°C overnight. Then, cells were washed with PBS (ice-cold). 400 *μ*L PI solution (5 *μ*g/mL) was added to the cells for 0.5 h in the dark. Then, cell cycle was analyzed by flow cytometers (Beckman Coulter, Brea, CA, USA).

### 2.10. Chromatin Immunoprecipitation

As described by Zhang et al. [30, 31], chromatin from cell cultures at 0 and 24 h was prepared. Chromatin was immunoprecipitated with 4 *μ*g of Sp1 antibody (Abcam, Britain). In parallel immunoprecipitation procedures, 4 *μ*g of G_aq_ antibody (Santa Cruz Biotechnology, USA) was used as a control. Precipitated DNA was amplified for 25 cycles. The promoter-specific forward primer is CTGTTTTCAGTGCCAACT, and the reverse primer is CATGGGGCCCCGTCGGCCGCTG.

### 2.11. Invasion Assay

Cells were suspended with a serum-free medium with the concentration of 2 × 10^5^/mL. We followed the methods of Wang et al. [32]. Nine random fields were counted under the microscope, and the statistical results were obtained.

### 2.12. ROS Analysis

Cells were suspended with a serum-free medium with the concentration of 2 × 10^5^/mL. We followed the methods of Schieber and Chandel [19]. Nine random fields were counted under laser scanning confocal microscopy, and the statistical results were obtained.

### 2.13. Statistical Analysis

Statistical analysis was conducted by SPSS 20.0 software (IBM). The data was presented as −*x* ± *s*. *t*-test was used for comparison between two groups. ANOVA was used for comparison among multiple groups. *P* < 0.05 were considered statistically significant.

## 3. Results

### 3.1. Sirt-1 Promotes Cell Proliferation of SK-N-SH Neuronal Precursor Cells

**Figure 1:**
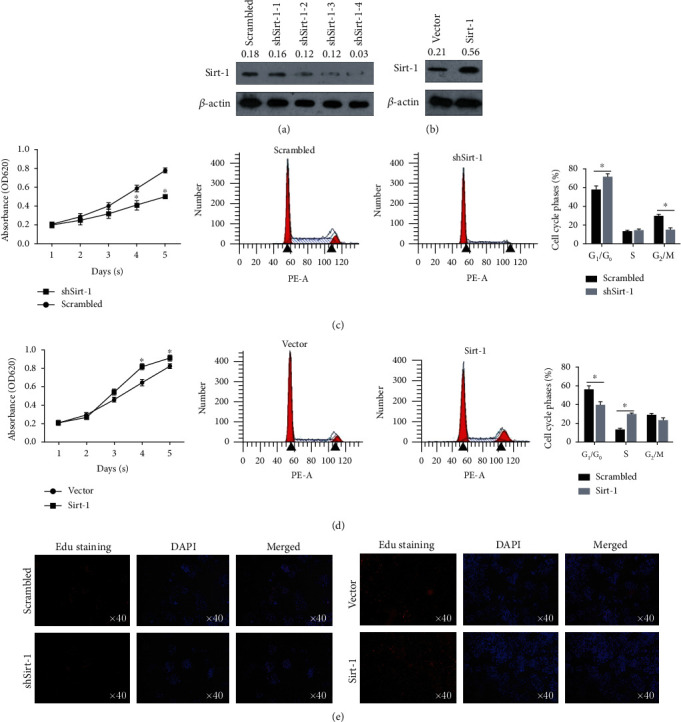
Sirt-1 positively promotes cell proliferation in SK-N-SH cells. (a) Stable knockdown of Sirt-1 by introducing shRNA targeting to Sirt-1 mRNA was performed, and knockdown efficacy was confirmed in four individual clones. (b) Stable overexpression of Sirt-1 was measured by semiquantitative western blot analysis to confirm the Sirt-1 protein level. After knockdown (c) or overexpression (d) of Sirt-1, cell viability from days 1-5 and cell cycle distribution were analyzed. ^∗^*P* < 0.05, vs. scrambled group. (e) EdU staining was then performed to further confirm the effect of Sirt-1 on the proportion of proliferating cells.

In order to study the possible functions of Sirt-1 protein in nerve cells/neural precursor cells, SK-N-SH cells were used. We tested the impact of altered Sirt-1 expression either by transient overexpression or by small RNA interference. Western blotting analysis validated that siRNA largely diminished Sirt-1 expression in SK-N-SH cells in a dose-dependent manner; in contrast, transient transfection resulted in a 2- to 3-fold increase in Sirt-1 protein expression (Figures 1(a) and 1(b)). Importantly, suppression of Sirt-1expression resulted in a significant reduction in SK-N-SH cell proliferation as analyzed by the CCK-8 method (Figure 1(c)). Conversely, overexpressing Sirt-1 dramatically enhanced the cell growth. When cell viability was significantly enhanced, proliferation-positive cells were increased, and Sirt-1 appears to promote SK-N-SH cell growth through facilitating their cell cycle progression because cells at G_1_/G_0_ were significantly reduced and those at the S or M phase were increased (Figure 1(e)), indicating that Sirt-1 affected the level of cell proliferation by regulating cell cycle. These results demonstrate that Sirt-1 is a positive regulator for neuronal cell proliferation and promotes cell growth through either enhancing cell cycle progression or inhibiting cell apoptosis or both.

### 3.2. Sirt-1 Selectively Inhibits the p53-p21 Pathway in SK-N-SH Cells

**Figure 2:**
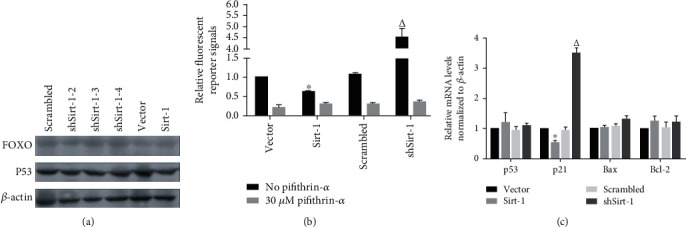
Sirt-1 regulates p53's transcriptional activity and its downstream gene. (a) After stable knockdown or overexpression of Sirt-1, FOXO and p53 levels were analyzed by performing western blot. (b) By performing fluorescent reporter assay, the effect of Sirt-1 on p53's transcriptional activity was measured. ^∗^*P* < 0.05, vs. vector group; ^△^*P* < 0.05, vs. scrambled group. (c) RT-qPCR was performed to detect mRNA levels of p53's downstream target genes, including p21, Bax, and Bcl-2. ^∗^*P* < 0.05, vs. vector group; ^△^*P* < 0.05, vs. scrambled group.

It has been shown that Sirt-1 regulates cell growth and survival through targeting p53 and FOXO1 [17]. We then speculated which pathway is regulated by Sirt-1 to promote SK-N-SH cell growth. The protein expression levels of p53 and FOXO1 were unaltered neither by Sirt-1 knockdown nor by its overexpression (Figure 2(a)). We then reasoned whether Sirt-1 regulates the transcription of p53 and FOXO1. Indeed, Sirt-1 expression inhibited, which its knockdown enhanced the luciferase activity driven by p53 (Figure 2(b)). As a control, the p53-specific inhibitor largely abolished p53 reporter activity (Figure 2(b)). Those results clearly indicate that Sirt-1 is a negative regulator of p53 transcriptional activity.

### 3.3. Sirt-1 Suppresses p53 Binding to p21 Promoter

**Figure 3:**
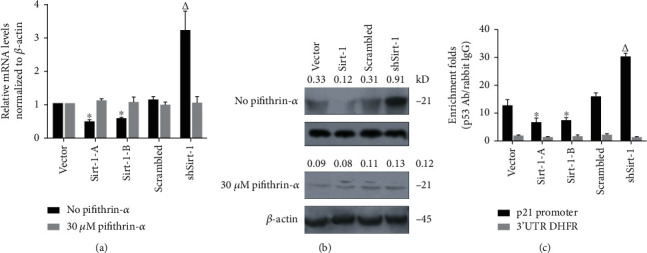
Sirt-1 indirectly regulates p21 depending on p53's transcriptional activity. p21 mRNA (a) and protein levels (b) were measured with or without the presence of 30 *μ*M pifithrin-*α*, an inhibitor of p53 transcriptional activity. ^∗^*P* < 0.05, vs. vector group; ^△^*P* < 0.05, vs. scrambled group. (c) Chromatin immunoprecipitation was performed to further confirm the effect of Sirt-1 on the binding of p53 to p21 promoter region. ^∗^*P* < 0.05, vs. vector group; ^△^*P* < 0.05, vs. scrambled group.

To determine whether Sirt-1 attenuates p21 mRNA transcription in a p53-dependent manner, we tested the effects of p53 inhibitor pifithrin-*α* pretreatment on Sirt-1-mediated p21 suppression. As expected, pifithrin-*α* pretreatment totally abolished the increase in p21 expression by Sirt-1 knockdown (Figures 3(a) and 3(b)). Together with the fact that neither p53 mRNA nor its protein expression levels were altered by Sirt-1, these results suggest that Sirt-1 inhibits p21 transcription possibly through a p53-dependent manner.

We then used chromatin immunoprecipitation to detect changes in the binding activity of p53 to the p21 promoter region after Sirt-1 overexpression/interference. 3′-UTR of DHFR was used as a target negative control. As was shown in Figure 3(c), the binding activity of p53 to p21 was decreased when interfered with Sirt-1 and increased when overexpression of Sirt-1.

### 3.4. Sirt-1 Exerts Its Deacetylation Activity on p53's Lysine 382 and Thus Regulates Cell Proliferation

**Figure 4:**
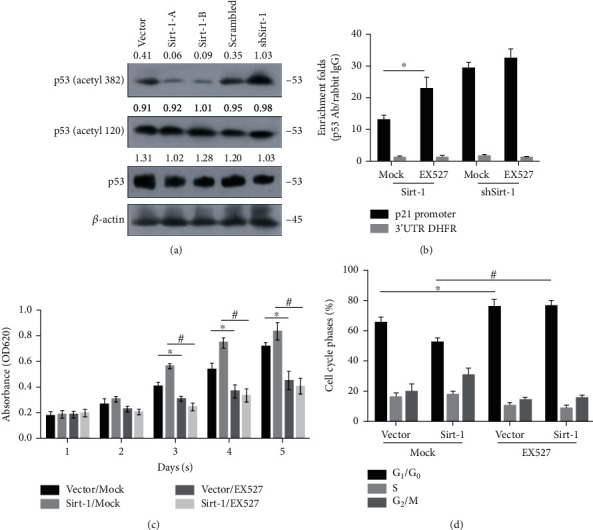
Sirt-1 regulates p53 acetylation at 382 amino acids (aa) and thus transcriptionally regulates p21. (a) Acetylation at 120 aa and 382 aa was detected by western blot. (b) Chromatin immunoprecipitation was performed after inhibiting acetylation by adding EX527. (c) Cell viability from days 1-5 was measured to access the effect of Sirt-1 via its acetylation activity. ^∗^*P* < 0.05, vs. vector group; ^#^*P* < 0.05, vs. scrambled group. (d) Cell cycle distribution was then measured by performing PI staining followed by flow cytometry. ^∗^*P* < 0.05, vs. vector group; ^#^*P* < 0.05, vs. scrambled group.

First, we examined whether the acetylation of p53 was regulated by Sirt-1, which had been known critical for p53 transcriptional activation [33]. The results showed that (Figure 4(a)), the expression of Sirt-1 had no effect on the total protein level of p53, but p53 deacetylation at 382 lysine was significantly inhibited (Figure 4(b)).

To determine how Sirt-1 regulates the binding of p53 to the promoter region of downstream target genes, we performed western blot to detect the acetylation level of p53 on 382 aa. p53 was highly acetylated at the lysine residue 382, which was largely inhibited by transient transfection of Sirt-1. Conversely, shRNA-mediated Sirt-1 knockdown resulted in a significant increase in p53 acetylation (Figure 4(a)). Since acetylation of p53 is required for its promoter binding activity, we then tested whether Sirt-1-mediated p53 deacetylation inhibited its promoter binding. Indeed, ChIP analysis detected a reduction in p53 promoter binding, which was reversed by Sirt-1-specific inhibitor EX7 (Figure 4(b)). In addition, Sirt-1 knockdown enhanced p53 promoter binding activity, further addition of Sirt-1 inhibitor, while slightly increased, but not statistically significantly enhanced p53 recruitment to the promoter (Figure 4(b)). Further analysis revealed similar changes of Sirt-1 catalytic activity in the cell lysate parallelly prepared as in Figure 4(b), suggesting regulation of p53 transcriptional activity relies on its deacylative activity (Figure 4(c)). More importantly, Sirt-1 enhanced, but its knockdown resulted in an inhibition of the cell cycle progression (Figure 4(d)). Collectively, these results suggest that Sirt-1 regulates cell cycle progression through deacetylating p53 to attenuate its transcriptional activity.

### 3.5. Sirt-1 Failed to Affect Cell Proliferation in SK-N-AS Cells

**Figure 5:**
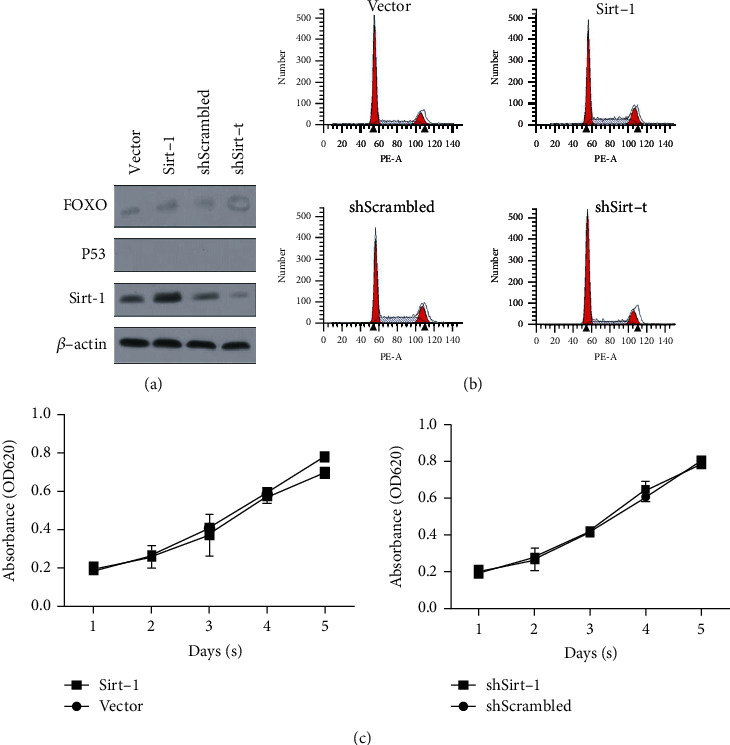
Sirt-1 failed to affect cell proliferation in SK-N-AS cells. (a) After overexpression or knockdown of Sirt-1, the protein levels of *β*-actin, Sirt-1, and FOXO were measured by western blot. (b) Cell viability from days 1-5 was measured to access the effect of Sirt-1 on SK-N-AS cells. (c) Cell cycle distribution was then measured by performing PI staining followed by flow cytometry. ^∗^*P* < 0.05, vs. vector group.

To further confirm whether Sirt-1 affects cell proliferation via regulating p53, SK-N-AS, a p53-null cell line was employed. Sirt-1 was overexpressed or knocked down in SK-N-AS. By performing western blot, the efficient overexpression of Sirt-1 and knockdown of endogenous Sirt-1 were confirmed (Figure 5(a)). Opposite to the effects of overexpressed or knockdown Sirt-1 in SK-N-SH, modification of Sirt-1 failed to obviously affect cell proliferation (Figures 5(b) and 5(c)) and cell cycle distribution (Figure 5(d)). Taken together, Sirt-1 affects cell proliferation and distribution of cell cycle mainly via the presence of p53.

### 3.6. Sirt-1 Affects Cell Migration and Invasion

**Figure 6:**
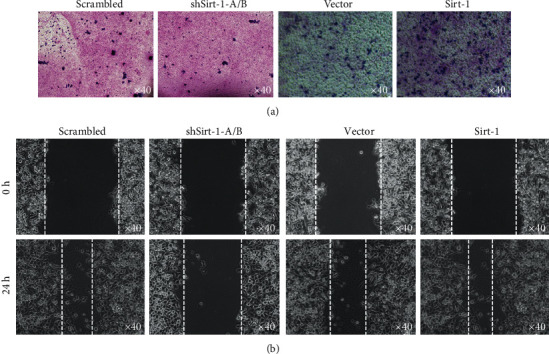
Sirt-1 affects cell migration and invasion. Knockdown or overexpression of Sirt-1, migration (a), and invasion (b) were measured.

Knockdown or overexpression of Sirt-1, migration, and invasion were measured. Migration results validated that siRNA largely diminished Sirt-1 expression, which dramatically weakened the migration (Figure 6(a)) and invasion (Figure 6(b)) of SK-N-SH cells. In contrast, transient transfection resulted in the increase in Sirt-1 protein expression, which dramatically enhanced the migration (Figure 6(a)) and invasion (Figure 6(b)) of SK-N-SH cells.

### 3.7. Sirt-1 Inhibits H_2_O_2_-Induced Neuronal Cell Death

**Figure 7:**
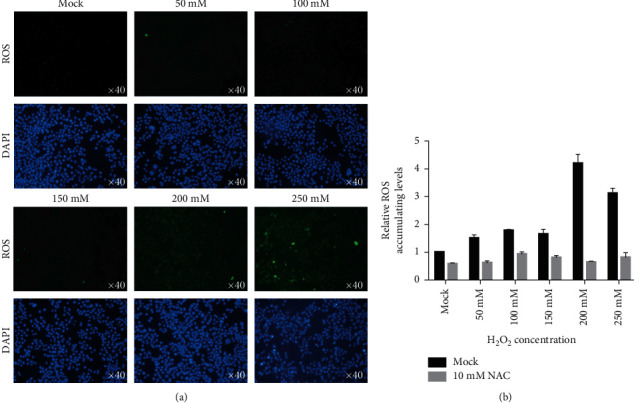
ROS level was induced after H_2_O_2_ treatment. (a) ROS staining was imaged after 24 h exposure to different concentrations of H_2_O_2_. (b) ROS level was quantitatively measured.

**Figure 8:**
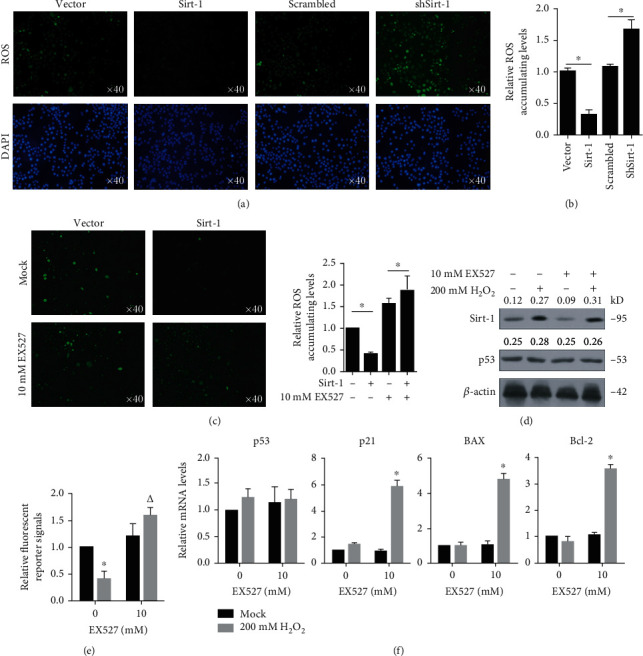
Sirt-1 abolished ROS accumulation after oxidative stress. (a, b) ROS accumulation of Sirt-1 was measured by the Reactive Oxygen Species Assay Kit under laser scanning confocal microscopy. The presence of Sirt-1 decreased ROS accumulation under oxidative stress. (c) The effect of Sirt-1 on the ROS level was measured. The results show that Sirt-1 affects the ROS level depending on the presence of its deacetylation activity. ^∗^*P* < 0.05. (d) The effects of adding EX527 on Sirt-1 and p53 expression were measured. Oxidative stress induced by H_2_O_2_ obviously increased Sirt-1 protein. (e) Transcriptional activity of p53 was measured after H_2_O_2_ treatment. ^∗^*P* < 0.05, vs. mock group; ^△^*P* < 0.05, vs. mock/EX527 group. (f) The effects of Sirt-1 to p53, p21, Bax, and Bcl-2 expression were measured. p53 and its downstream target gene expressions were measured by RT-qPCR. ^∗^*P* < 0.05, vs. mock/EX527 group.

In addition to p53, it has been shown that Sirt-1 functions as a negative regulator for ROS production, implying a possibility that Sirt-1 may protect neurons from oxidative stress-induced cell death. After 24 h treatment with 50-250 mM H_2_O_2_, reactive oxygen species (ROS) was obviously increased and scavenged by adding 10 mM of NAC (Figures 7(a) and 7(b)), and thus, 200 mM of H_2_O_2_ was added to induce oxidative stress. Overexpressed Sirt-1 scavenged ROS production in SK-N-SH with H_2_O_2_, and knockdown of Sirt-1 presented opposite effect (Figures 8(a) and 8(b)). Similarly, the addition of 10 mM EX527 increased ROS accumulation, which indicated that the deacetylation activity of Sirt-1 is critical for its ROS suppressive activity (Figure 8(c)). Notably, H_2_O_2_ treatment obviously increased the Sirt-1 protein level without affecting p53 protein (Figure 8(d)) and decreased p53's transcriptional activity (Figure 8(e)). Our results demonstrated that oxidative stress induced Sirt-1 in neuron cells, possibly to protect them from oxidative stress-induced cell death, through suppressing p53 transcriptional activity (Figure 8(f)).

**Figure 9:**
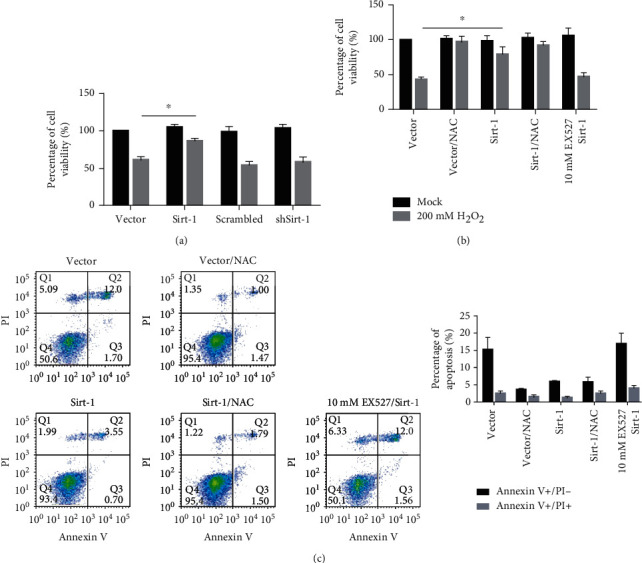
Sirt-1 protects cells from oxidative stress induced by H_2_O_2_ via its deacetylation activity. (a) Adding 200 mM H_2_O_2_, the percentage of cell viabilities in the vector group, Sirt-1 group, scrambled group, and shSirt-1 group were measured. Sirt-1 presence protects cells from H_2_O_2_-induced decrease in cell viability. ^∗^*P* < 0.05, vs. vector group. (b, c) The percentage of apoptosis in the vector group, Sirt-1 group, scrambled group, shSirt-1 group, and EX527 group was measured. Sirt-1 exerts protective effects on cell viability and cell survival from apoptotic cell death depending on its deacetylation activity.

To evaluate the protective effect of Sirt-1 from oxidative stress-induced SK-N-SH cell death, we first detect cell viability after 48-hour incubation in 200 mM H_2_O_2_. As it is shown in Figure 9(a), overexpressed Sirt-1 significantly improved cell viability under oxidative stress. Notably, knockdown of Sirt-1 failed to affect cell viability, possibly due to the endogenous Sirt-1 expression that remains at a low level and barely affected cell injury induced by oxidative stress. Consistent with this, inhibition of deacetylation activity of endogenous Sirt-1 by EX527 did not show any effects (Figure 9(b)). Taken together, our results show that overexpressed Sirt-1 inhibits oxidative stress-induced apoptotic cell death depending on its deacetylation activity (Figure 9(c)).

## 4. Discussion

Sirt-1 has been widely studied as a regulator of many cellular physiological and pathological processes [34, 35] It was also observed that Sirt-1 was associated with cognitive impairment in AD, and similar results were obtained in depression studies [36, 37]. However, how Sirt-1 affected AD or depression is still unknown, especially the association among Sirt-1, AD, and depression. In this study, we investigated the regulation of Sirt-1 on the proliferation in SK-N-SH cells and found that Sirt-1 affected physiological processes. Modified Sirt-1 by overexpression and knockdown transcriptionally regulated p53's downstream target gene, p21, in a p53-dependent manner. Sirt-1 regulates the transcriptional activity of p53 by inhibition of the acetylation of p53. By employing human neuroblastoma cell line SK-N-AS, which is a p53-null cell line, upregulation or downregulation of Sirt-1 failed to affect p21 and cell cycle distribution, which further confirmed the necessity of p53 on Sirt-1's regulatory roles in Figure 2(b). The deacetylation activity of Sirt-1 is necessary to regulate physiological processes, including cell proliferation via regulating the transcriptional activity of p53, and thus regulates its downstream target genes. The above results suggest that Sirt-1 may be closely related to the raising of AD by exerting protective effects on neuronal cells.

We also found that Sirt-1 promotes cell proliferation via regulating cell cycle progression. Overexpression of Sirt-1 enhanced cell viability and cell proliferation by promoting the entry of cell cycle from the G_1_ phase to the G_0_ phase, which is consistent with previous studies [38, 39]. As an important member of the sirtuin family, Sirt-1 is involved in regulating cell proliferation in many human cells. In AD model animals, overexpression of Sirt-1 slowed cell death, neurodegeneration, and cognitive decline [40, 41]. Acetylshikonin, which upregulates Sirt-1, can reduce cognitive dysfunction [42]. In contrast, inhibition of Sirt-1 expression can prevent oligodendrocyte progenitor cell proliferation [43]. Downregulation of Sirt-1 in senescent microglia plays a key role in aggravating senescence in mice, while overexpression of Sirt-1 significantly prolongs life [44].

We observed that in SK-N-NH, but not in SK-N-AS, inhibition of Sirt-1 promoted the transcriptional activity of p53, but not that of FOXO1, which indicated that Sirt-1 specifically regulates p53 in SK-H-SH with wild-type p53. p53 is one of the most critical transcriptional factors to regulate nonhistone proteins [45, 46]. Acetylation of p53 increases the stability of the p53 protein and its antiviral to DNA damage, which is necessary for the detection of DNA damage and the activation of oncogenes [47, 48]. Previous literature studies have shown that Sirt-1 regulates the transcriptional regulatory activity of p53 by deacetylation of p53, which is similar to our results [20].

Under oxidative stress, p53 promotes cell death after the failure of DNA repair [17]. Sirt-1 was reported to exert protective effects on rat neuronal cells in vivo by inhibiting p53 transcriptional activity. EX527, a Sirt-1 inhibitor, exerted similar effects to that of Sirt-1 knockdown. EX527 treatment potentially due to the induction of ROS by oxidative stress could be scavenged by activation of Sirt-1 and inhibition of p53 [19]. By considering that oxidative stress is recognized as a central stress factor in major depressive disorder (MDD) and AD, activation of Sirt-1 may prevent from MDD and AD via protecting neuronal cells from oxidative stress.

In summary, we demonstrated the important role of Sirt-1 in maintaining the proliferation and suppressing the oxidative stress-induced neuronal cell death. At the molecular level, Sirt-1 inhibits p53 transcription activation through directly deacetylating p53 and thus reduces p21 gene expression. On the other hand, Sirt-1 protects neuronal cells from oxidative stress-induced apoptosis via suppressing ROS production. These results provide a molecular explanation of the protective effect of Sirt-1 on AD and depression-related diseases and provide a rationale for Sirt-1 as a biomarker and therapeutic target for the diagnosis, treatment, and prevention of AD and depression-related diseases.

## Data Availability

The data used to support the findings of this study are available from the corresponding author upon request.
